# A method to correct for population structure using a segregation model

**DOI:** 10.1186/1753-6561-3-s7-s104

**Published:** 2009-12-15

**Authors:** Qingfu Feng, Joseph Abraham, Tao Feng, Yeunjoo Song, Robert C Elston, Xiaofeng Zhu

**Affiliations:** 1 Department of Epidemiology and Biostatistics, Case Western Reserve University, Cleveland, Ohio 44106 USA

## Abstract

To overcome the "spurious" association caused by population stratification in population-based association studies, we propose a principal-component based method that can use both family and unrelated samples at the same time. More specifically, we adapt the multivariate logistic model, which is often used in segregation analysis and can allow for the family correlation structure, for association analysis. To correct the effect of hidden population structure, the first ten principal-components calculated from the matrix of marker genotype data are incorporated as covariates in the model. To test for the association, the marker of interest is also incorporated as a covariate in the model. We applied the proposed method to the second generation (i.e., the Offspring Cohort), in the Genetic Analysis Workshop 16 Framingham Heart Study 50 k data set to evaluate the performance of the method. Although there may have been difficulty in the convergence while maximizing the likelihood function as indicated by a flat likelihood, the distribution of the empirical *p*-values for the test statistic does show that the method has a correct type I error rate whenever the variance-covariance matrix of the estimates can be computed.

## Background

To overcome a potential problem ("spurious" association) caused by population stratification in population-based association studies, several methods have been proposed recently. These approaches are favored because unrelated case-control studies are considered more powerful and easier for collecting DNA samples than family-based studies.

The "Genomic Control" (GC) method adjusts the standard chi-square statistic *X*^2 ^to *X*^2^/*λ*, where *λ *can be estimated by using genomic marker data, and the adjusted statistic follows a chi-square distribution [1-4]. When the sample arises from a population in which population structure exists, the ordinary chi-square statistic may follow a noncentral chi-square distribution *X*^2^(*δ*) with noncentrality parameter *δ*. The GC method is dependent on the estimate of *λ*, which is dependent on the markers selected for controlling the effect of population stratification and may result in either a conservative or liberal test statistic [5,6].

Another approach, named "Structured Association" (SA), is a Markov-chain Monte Carlo (MCMC)-based method that uses independent genomic markers to infer the number of subpopulations and the ancestry probabilities of individuals from putative unstructured subpopulations, and this inferred information is further used in the test for association [5,7]. The method was also extended to inferring the population structure while simultaneously estimating the model parameters and testing for association [8]. However, when the number of subpopulations is large, the SA method might be computationally intensive.

Recently, principal-component analysis (PCA)-based methods, which calculate the principal components of marker genotype data to represent the genetic background, have been widely used in association studies [6,9-13]. Specifically, Price et al. [12] proposed a method of regressing both the phenotype and marker genotype values on the principal components for unrelated data, and association between the phenotype and the marker is tested by using the residual correlation. More recently, Zhu et al. [13] extended the method by allowing both family and unrelated samples in the association test while correcting for population stratification.

In this report, we propose a method to test the association between a binary trait and a marker by using a segregation model that allows for the family correlation structure. We apply an idea similar to Zhu et al.'s [13] to correct for the effect of population stratification. We apply the method to the Genetic Analysis Workshop 16 (GAW16) Framingham Heart Study 50 k data set to evaluate the performance of the method.

## Methods

We use a regression model to specify a phenotype as a function of the genotype of a single nucleotide polymorphism (SNP) of interest. Because our focus is on binary traits, we apply the usual approach of logistic regression. We summarize the genotype data by a principal-component analysis to extrapolate axes of genetic variation that are defined as the top eigenvectors of a covariance matrix between samples. In data sets with population structure, axes of variation often have a geographic interpretation [12]. Thus, incorporating principal components in the model can reduce the effect caused by population stratification by adjusting the logit by an amount attributable to ancestry along each axis.

### PCA

Consider samples that include both family and unrelated individuals. For simplicity, we only consider nuclear families. Suppose our data contain *N*_*f *_nuclear families, *N*_*d *_unrelated cases, and *N*_*c *_unrelated controls. For nuclear families, assume there are *k*_*i *_members in the *i*^th ^family with the first two (*j *= 1 or 2) being the father and mother. Then we have in total  individuals. Again for simplicity, we assume there are *N *families with *k*_*i *_= 1 if *i *> *N*_*f*_. In this way we define each unrelated case or unrelated control as a separate family of size one. That is, we have *N *= *N*_*f *_+ *N*_*d*_+ *N*_*c *_families. Let *y*_*ij *_be the binary trait value, taking on the value 0 if unaffected and 1 if affected, for the *j*^th ^individual in the *i*^th ^family. Let *g*_*ij *_be the marker genotypic value of the *j*^th ^individual in the *i*^th ^family, coded according to an additive mode of inheritance. *M *diallelic markers are genotyped. Let *X*_*ij *_= (*x*_*ij*1_, *x*_*ij*2_, ..., *x*_*ijM*_) be a column vector of the marker genotypic values for the *j*^th ^individual in the *i*^th ^family with *x*_*ijl *_= 0,1, or 2, corresponding to a homozygote, the heterozygote, and the other homozygote, *l *= 1,2, ..., *M *We perform a PCA to summarize the marker data, but for this we only use unrelated individuals, i.e., the parents in each family and the unrelated cases and controls, because a naive PCA with all the available data will result in biased directions of maximum variability for the data. Let

denote the variance-covariance matrix of the marker data for these unrelated individuals in our data, where  denotes the overall mean of *X*. Let *e*_*l *_be the *l*^th ^eigenvector corresponding to the *l*^th ^largest eigenvalue of Σ, *l *= 1, 2, ..., *M*. The *l*^th ^principal component for individual *j *of family *i*, *t*_*ijl*_, can be calculated by *t*_*ijl *_= (*X*_*ij *_- )^*T*^*e*_*l*_, where *i *= 1, 2, ..., *N*, *j *= 1, 2, ..., *k*_*i*_, and *i *= 1, 2, ..., *M*. In this study we only consider the first *L *= 10 principal components, as suggested by Zhu et al. [13] and Price et al. [12] in our analysis.

### A multivariate logistic model

Now we formulate the relationship between a binary trait *y*_*ij *_and a marker genotypic value of interest, *g*_*ij*_, by the following logistic model:

where *β*_0 _is the intercept, *β*_*g *_represents the effect of the genotype on the trait, and *β*_1_, ..., *β*_*L *_are the coefficients for the first *L *principal components, which are used to eliminate the effect caused by the population stratification. We also introduce an index variable *I*, which is defined as *I*_*ij *_= 1 if an individual's markers are used to calculate the principal components, and *I*_*ij *_= 0 otherwise. This model can allow us to incorporate any other covariates. The likelihood function of the multivariate logistic model (MLM) of the *i*^th ^family is [14]:

where *B*_*ij *_= *β*_0 _+ *β*_*g*_*g*_*ij *_+ *β*_1_*t*_*ij*1_+ *β*_*L*_*t*_*ijL *_+ *β*_*I*_*I*_*ij*_, and *δ*_*i*1_, *δ*_*i*2_, *δ*_*i*3 _and *δ*_*i*4 _are parameters that, respectively, measure the association between parent 1 and offspring, parent 2 and offspring, sib-pairs, and spouse pairs. These *δ*_*i *_values take values in the range:  for all *B*_*ij*_, *B*_*il *_and .

The overall likelihood *L *is the product of the likelihoods for all families,  We consider a marker and the principal components as covariates. To estimate the unknown parameters, we used the program SEGREG in S.A.G.E. [15], which is based on maximum likelihood estimate (MLE) methods. For simplicity, we set *δ*_*i*4 _= 0, i.e., husband and wife were assumed to be independent, and we further assumed all the remaining *δ *values are the same. Because our null hypothesis is no association, *H*_0_: *β*_*g *_= 0, rather than testing for a major gene, we maximized the likelihood function under the no major gene model, i.e., we assumed a single multivariate logistic distribution rather than a mixture of such distributions, which would be necessary for segregation. We used the Wald statistic to test the null hypothesis.

### Application to the GAW16 Framingham Heart Study 50 k data set

We applied our method to the GAW16 Framingham Heart Study 50 k data set. We only used the second generation, i.e., the Offspring Cohort. The founders of each family were included to calculate the principal components. If no founder of a family was available, we randomly chose one individual. The spouses of the Offspring were treated as unrelated individuals. Hypertension was defined based on the data at Examination One. We defined an individual as affected if his/her systolic blood pressure was greater than or equal to 140 mm Hg, or diastolic blood pressure was greater than or equal to 90 mm Hg, or he/she was on medication, and unaffected otherwise.

## Results

### Quality control

The GAW16 50 k Offspring Cohort Data Set includes 3,850 individuals. Among them, 1,170 individuals were not genotyped and were not used for further analysis. Of the remaining 2,680 individuals, 89 were under the age of 18 and they were also removed because our analyzed phenotype is hypertension. There are 48,028 markers available, of which 6,051 have a missing genotyping rate of over 10% and 8,163 have minor allele frequencies less than 5%. Those 14,214 markers were dropped from any further analysis. Our analysis results were thus based on 2,591 individuals and 33,811 markers.

### The performance of the method

**Figure 1:**
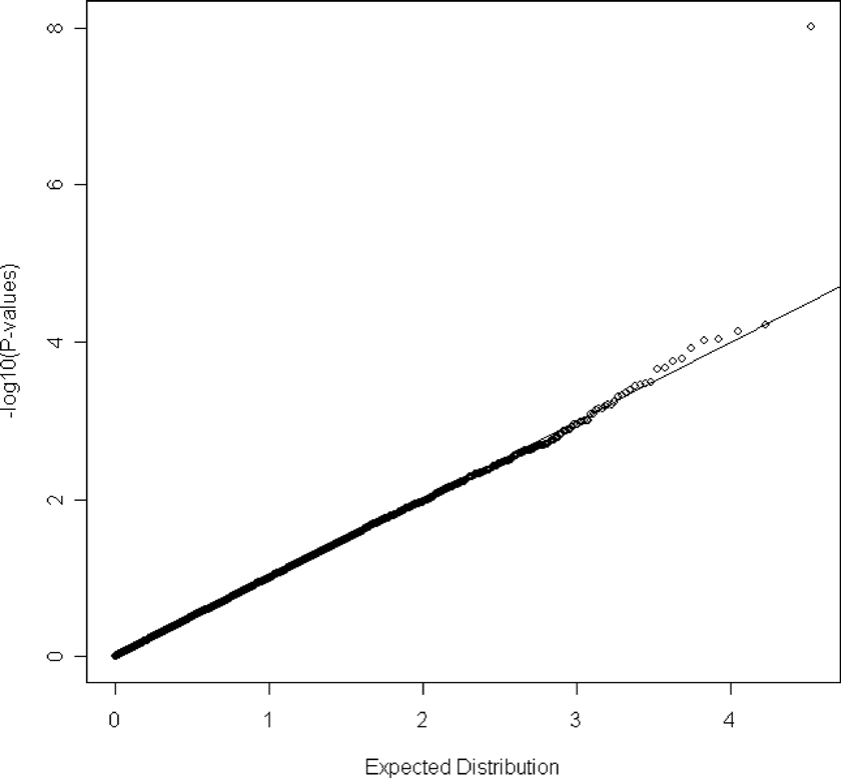
**The Q-Q plot for the empirical *p*-values in the model without incorporating age, sex, and BMI**. -log_10_(*p*-value) is plotted against its expected value under the null hypothesis. A 45-degree line would be expected if the results are due to chance alone.

**Figure 2:**
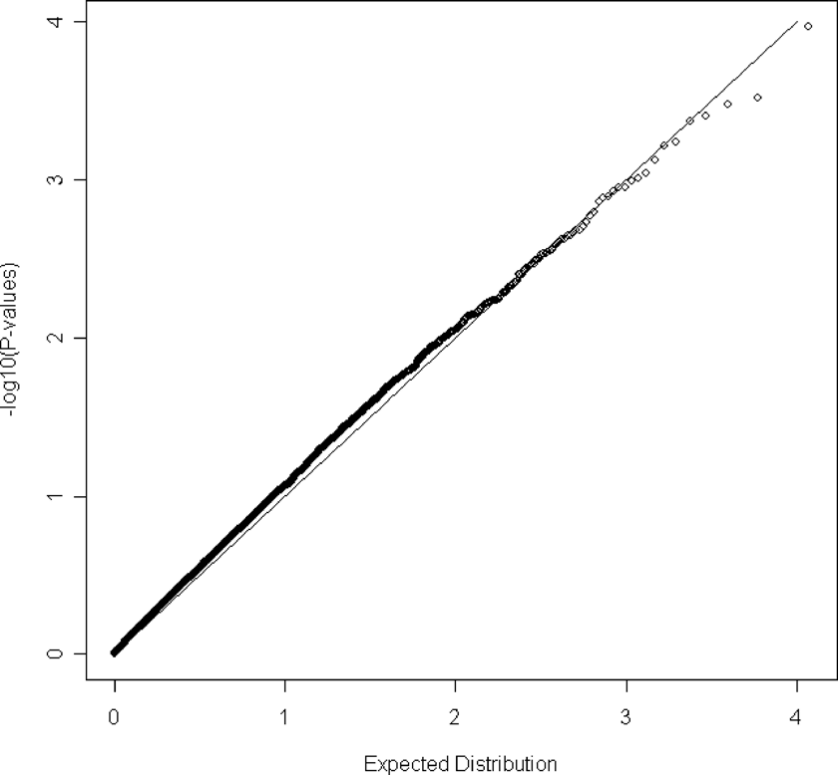
**The Q-Q plot for the empirical *p*-values in the model incorporating age, sex and BMI**. -log_10_(*p*-value) is plotted against its expected value under the null hypothesis. A 45-degree line would be expected if the results are due to chance alone.

We calculated the principal components for each individual using the remaining 33,811 markers using the software FamCC [13]. The *p*-values of the association test for all the SNPs were obtained using the software SEGREG in S.A.G.E. [15]. We first used the model without incorporating age, sex, and body mass index (BMI). We identified seven markers whose maximization of the likelihood function may not have converged using SEGREG and their *p*-values are not reported. The quantile-quantile (Q-Q) plot of the *p*-values for the rest of markers can be found in Figure 1. The observed distribution of *p*-values does not depart from that under the null hypothesis, suggesting the proposed method has a reasonable type I error rate. Notice that there is one SNP, rs4084639, standing out alone from the rest of the *p*-values. We next incorporated age, sex, and BMI as covariates in the model. However, this created a flat likelihood, so it is possible that non-convergence of the maximization in SEGREG occurred for 22,027 markers. The Q-Q plot of the *p*-values for the markers with definite converged maximization is presented in Figure 2. This Q-Q plot does not show any substantial departure, suggesting the type I error rate is reasonably controlled. The most significant marker (rs4084639) in Figure 1 is now no longer significant. Further correlation analysis between rs4084639 and age, sex, and BMI indicates rs4084639 is highly correlated with sex (correlation coefficient = 0.99), suggesting that the highly significant association between this SNP and hypertension identified in the first model is due to the strong correlation between hypertension and sex. However, we did not screen for Hardy-Weinberg equilibrium. A Hardy-Weinberg equilibrium screen would have eliminated the marker rs4084639 from consideration. Furthermore, most males were CG heterozygotes and most females were GG homozygotes. A manual BLAST analysis shows hits to both chromosome 1 and chromosome Y, suggesting that this marker may amplify two non-polymorphic regions in males and one non-polymorphic region in females. Although SEGREG suggested the possibility of non-convergence for many of the markers, when convergence was verified, type I error was well controlled. To find the cause of possible non-convergence problem in SEGREG, we repeated our analyses after relaxing the convergence criteria in SEGREG and fixing the ten principal-component covariate coefficients at their values estimated under the null hypothesis. This had little effect on the results; the likelihood was still flat, indicating that the data are insufficient to estimate all the covariates together with the familial correlations.

## Discussion

We proposed a novel association method that adopts the idea of dealing with residual family correlations as has been used for a segregation model for binary traits, but without using the usual mixture distribution that is an essential part of segregation analysis. Meanwhile, the proposed method also incorporates the marker principal components for controlling the effect of population stratification in family data, as proposed by Zhu et al. [13]. One advantage of the proposed method is the flexibility of incorporating different kinds of family correlation structure. Although SEGREG suggested the possibility of non-convergence for many of the markers in this study, when convergence was verified (i.e. there was no difficulty in estimating the variance-covariance matrix of the estimates), type I error was well controlled. It is well known that when the number of parameters to estimate is large, the likelihood function can be flat around the MLE, as we found in our analysis when we added age, sex, and BMI as covariates, and that there is an increased computational burden (a single maximization needed 6 minutes for the full model, while it needed only 3 minutes when age, sex, and BMI were not incorporated, on the Intel Xeon 1.6 GHz cluster). Although we described our methods using nuclear families only, the methods could be generalized in an obvious way to extended pedigrees. Indeed, the families in the GAW16 Framingham Heart Study 50 k data set Offspring Cohort (the second generation) were not nuclear families, but rather a type of extended pedigrees. Hence, our results could be thought of as pertaining to extended pedigrees plus unrelated cases and controls. Another program, UNPHASED [16], also analyzes similar types of data, though its focus is on haplotype data. This method does not handle population stratification directly, but could do so in a similar manner. The models in UNPHASED could incorporate covariates. For instance, one could incorporate the principal components in the model as "confounders" to adjust for the population stratification. We used the first ten principal components in our analysis. In the case that a population is admixed with a relatively small number of ancestral populations, ten principal components might be excessive. On the other hand, in the case that a population is admixed with a relatively large number of ancestral populations, ten principal components might not be enough. However, we can test whether a principal component is significant in the model. If not, we drop it.

We only applied the proposed model to the GAW16 Framingham Heart Study 50 k data set. Because the underlying disease model is unknown we are unable to evaluate the power of the proposed method. Future studies will also include power comparison with the method proposed by Zhu et al. [13] as well as type I error analysis under different admixed population samples using simulations.

## Conclusion

We propose an association method using a segregation analysis based model to deal with family structure while controlling for population structure. By analyzing a real data set from the GAW16 Framingham Heart Study, we showed that the method performs well in the sense of controlling type I error rate, whenever we can be sure that the maximization of the likelihood function is successful.

## List of abbreviations used

BMI: Body mass index; GAW16: Genetic Analysis Workshop 16; GC: Genomic control; MCMC: Markov-chain Monte Carlo; MLE: Maximum likelihood estimate; PCA: Principal-component analysis; Q-Q: Quartile-quartile; SA: Structured association.

## Competing interests

The authors declare that they have no competing interests.

## Authors' contributions

XZ and JA designed the methods. QF, TF, and YS performed the data quality control. QF analyzed the data. QF, XZ, and RCE interpreted the results and wrote the paper. All authors read and approved the final manuscript.
